# Private sector engagement in the COVID-19 response: experiences and lessons from the Democratic Republic of Congo, Nigeria, Senegal and Uganda

**DOI:** 10.1186/s12992-022-00853-1

**Published:** 2022-06-15

**Authors:** Steven N. Kabwama, Suzanne N. Kiwanuka, Mala Ali Mapatano, Olufunmilayo I. Fawole, Ibrahima Seck, Alice Namale, Rawlance Ndejjo, Susan Kizito, Fred Monje, Marc Bosonkie, Landry Egbende, Segun Bello, Eniola A. Bamgboye, Magbagbeola D. Dairo, Ayo S. Adebowale, Mobolaji M. Salawu, Rotimi F. Afolabi, Issakha Diallo, Mamadou M. M. Leye, Youssou Ndiaye, Mane Fall, Oumar Bassoum, Tobias Alfvén, William Sambisa, Rhoda K. Wanyenze

**Affiliations:** 1 Department of Community Health and Behavioral Sciences, Makerere University School of Public Health, Kampala, Uganda; 2 Department of Global Public Health, Karolinska Institutet, Stockholm, Sweden; 3 Department of Health Policy Planning and Management, Makerere University School of Public Health, Kampala, Uganda; 4 Kinshasa School of Public Health, Kinshasa, Democratic Republic of Congo; 5 Faculty of Public Health, College of Medicine, University of Ibadan, Oyo, Nigeria; 6 Department of Preventive Medicine and Public Health, University Cheikh Antar Diop, Dakar, Senegal; 7 Department of Disease Control and Environmental Health, Makerere University School of Public Health, Kampala, Uganda; 8 Makerere University School of Public Health, Kampala, Uganda; 9 Bill and Melinda Gates Foundation, Seattle, USA

**Keywords:** COVID-19, Private sector, COVID-19 testing, Public–private partnerships

## Abstract

**Background:**

Private entities play a major role in health globally. However, their contribution has not been fully optimized to strengthen delivery of public health services. The COVID-19 pandemic has overwhelmed health systems and precipitated coalitions between public and private sectors to address critical gaps in the response. We conducted a study to document the public and private sector partnerships and engagements to inform current and future responses to public health emergencies.

**Methods:**

This was a multi-country cross-sectional study conducted in the Democratic Republic of Congo, Nigeria, Senegal and Uganda between November 2020 and March 2021 to assess responses to the COVID-19 pandemic. We conducted a scoping literature review and key informant interviews (KIIs) with private and public health sector stakeholders. The literature reviewed included COVID-19 country guidelines and response plans, program reports and peer-reviewed and non-peer-reviewed publications. KIIs elicited information on country approaches and response strategies specifically the engagement of the private sector in any of the strategic response operations.

**Results:**

Across the 4 countries, private sector strengthened laboratory systems, COVID-19 case management, risk communication and health service continuity. In the DRC and Nigeria, private entities supported contact tracing and surveillance activities. Across the 4 countries, the private sector supported expansion of access to COVID-19 testing services through establishing partnerships with the public health sector albeit at unregulated fees. In Senegal and Uganda, governments established partnerships with private sector to manufacture COVID-19 rapid diagnostic tests. The private sector also contributed to treatment and management of COVID-19 cases. In addition, private entities provided personal protective equipment, conducted risk communication to promote adherence to safety procedures and health promotion for health service continuity. However, there were concerns related to reporting, quality and cost of services, calling for quality and price regulation in the provision of services.

**Conclusions:**

The private sector contributed to the COVID-19 response through engagement in COVID-19 surveillance and testing, management of COVID-19 cases, and health promotion to maintain health access. There is a need to develop regulatory frameworks for sustainable public–private engagements including regulation of pricing, quality assurance and alignment with national plans and priorities during response to epidemics.

## Introduction

The Corona Virus Disease 2019 (COVID-19) pandemic is by far the biggest and most devasting public health crisis of the new millennium. It has caused significant disruption of global and national economies, and reversed years of investment in economic and social capital development. The pandemic has overwhelmed health systems, particularly in low- and middle income countries (LMICs) where the provision of essential services for communicable and noncommunicable diseases including mental health, maternal and child health services as well as nutrition services have been significantly disrupted [1, 2]. The pandemic has blatantly exposed the inability of the public sector to independently deliver on global and national development and health system goals [3]. Recent literature has also shown that there is a multiplicity of factors that explain the variation in COVID-19 disease burden on health systems [4].

The engagement of the private sector in health systems strengthening and service delivery is important in augmenting public health sector efforts and scarce resources in improving the health outcomes and well-being. In their guidance on developing national action plans for health security, the World Health Organization (WHO) emphasized that both public and private entities from multiple sectors including health, agriculture, environment, security, emergency management, education and transportation should work hand-in-glove to support their nation’s development of capacities for health security [5]. Furthermore, the WHO provided a framework for governance that will facilitate the private sector to contribute to the achievement of health security and Universal Health Coverage (UHC) [6]. The private sector is broadly defined and differs across domains of interest, contexts and countries. For instance, the Organization for Economic Cooperation and Development (OECD) glossary of statistical terms defines the private sector as "private corporations, households and non-profit institutions serving households [7]”. It includes large multinationals to micro-enterprises, SMEs to cooperatives, and even informal economy structures. Within the health sector the WHO defines the private health sector as all institutions and individuals that are neither owned nor directly controlled by governments and are involved in the provision of health services including for-profit, not-for-profit, formal, informal, domestic and international entities [8]. For the purposes of this article we will adopt both definitions to include corporations and non-profit institutions as well as individuals.

The Global Health Security Index (GHSI), a preparedness and response framework used to assess country capacities for responding to public health emergencies, reported that only 11% of 195 countries assessed using this framework had established formal ways to engage the private sector in public health emergency preparedness and response [9]. In most countries therefore, civil society organizations, for-profit and not-for-profit entities as well as multi-lateral organizations have engaged in the COVID-19 response to curtail the spread of the virus and ensure continued access to essential services. The engagement and participation models of the private sector with the public sector in the response to the COVID-19 pandemic in LMICs and in Sub-Saharan Africa is yet to be documented. There are knowledge gaps and limited documentation of models adopted for effective implementation of public–private sector partnerships that fully leverage the private sector involvement in healthcare service delivery including health systems strengthening [10] including epidemic preparedness and response in particular. We conducted a multi-country study to document the procedures, policies and strategies for responding to the COVID-19 pandemic adopted in four countries in Sub-Saharan Africa, namely in the Democratic Republic of Congo, Nigeria, Senegal, and Uganda. The experiences and lessons are relevant currently as countries respond to COVID-19 and roll out vaccination as well as deal with future public health emergencies.

## Methods

### Study area

This was a multi-country study conducted in the Democratic Republic of Congo (DRC), Nigeria, Senegal and Uganda to assess and document country responses to the COVID-19 pandemic in sub-Saharan Africa. Countries were included in the study because of their historical experience in managing public health emergencies of international concern [11–14] and a variation in the COVID-19 responses [15] to provide an opportunity for learning across countries.

### Study countries

Nigeria and Senegal are located in West Africa, the Democratic Republic of Congo in Central Africa and Uganda in East Africa.

All 4 countries had coordination structures that allowed for a multi-sectoral engagement in the response including non-governmental organizations and international organizations such as WHO. The DRC had a multisectoral response committee (Comite multisectoriel de reposte) that was headed by the prime minister and with a secretariat at the Ministry of Health. In Nigeria, there was a Presidential Taskforce that included ministries and government agencies, and an inter-ministerial multi-sectoral technical working group with a secretariat at the Federal Ministry of Health. Senegal had a National Epidemic Management Committee (CNGE) that allowed for multi-sectoral engagement. Uganda had a National Taskforce that was headed by the President and included various ministries as well as a strategic level committee.

### Study design

The study employed qualitative methods to obtain information about the engagement of the private sector in the COVID-19 response across the four countries in the DRC, Nigeria, Senegal and Uganda. For the purposes of this paper, we focused on the engagement of health and non-health for-profit and not-for-profit, formal, informal, domestic or international organizations in any of the strategic preparedness and response operations of the COVID-19 response [16].

### Data collection

The data collection started with a review of literature which was then supplemented with interviews with key informants. Data collection for both the literature review and qualitative interviews across the four countries started in November 2020 and was concluded by March 2021.

### Literature review

The literature review focused on studying each country’s COVID-19 country guidelines and response plans, program reports, websites, meeting minutes and presentations. The review also involved a search and curation of peer-reviewed and non-peer-reviewed publications describing the COVID-19 response in general and the involvement of the private sector either individually and/or collectively with the public sector in each country’s response to COVID-19 epidemic.

### Key informant interviews

Key informant interviews (KIIs) were conducted to understand and document country COVID-19 response approaches, practices, and strategies with the engagement of the private sector in as one of the key thematic areas. Overall, the KIIs were conducted to elicit information on each country’s response including COVID-19 response coordination, COVID-19 surveillance and contact tracing, laboratory testing, case management and continuity of essential services. Specifically, this study focuses on findings related to the involvement of the private sector in COVID-19 strategic preparedness and response operations including coordination, risk communication and community engagement, surveillance, points of entry, rapid response teams, national laboratory systems, infection prevention and control, case management or logistics, procurement and supply management [16]. Key informants included health managers at various levels of the health system, health facility staff such as nurses and midwives, policy makers at the national level, and community health workers. In the DRC, 22 interviews were conducted, in Nigeria, 32 interviews were conducted, in Senegal, 21 interviews were conducted and in Uganda, 35 interviews were conducted. The interviews were conducted either over the phone, using internet electronic communication platforms like Zoom™ or in-person in strict observance of COVID-19 standard prevention operating procedures like physical distancing and mask wearing.

### Data management and analysis

Findings from the literature review about the engagement of the private sector were categorized using the building blocks of a health system [17] as the guiding framework and further presented by the specific strategic preparedness and response operations of the response [16].

All interviews were audio recorded and transcribed verbatim. The interview transcripts were read to generate a codebook based on the broad themes of the study objectives. The coding was done in a computer program for data analysis (Atlas.ti). The codes were then resynthesized into subthemes. Results were presented as quotes to supplement findings from the literature review about the engagement of the private sector in the COVID-19 response.

### Ethical considerations

This work was part of the Bill and Melinda Gates Foundation and Gates Venture funded “Assessment of the COVID-19 Response in the DRC, Nigeria, Senegal and Uganda” project for which each participating country obtained ethical approval to conduct the study. In Uganda, the study obtained ethical approval from the Makerere University School of Public Health Higher Degrees Research and Ethics Committee (HDRC #903) and was registered with the Uganda National Council for Science and Technology (UNCST #HS1121ES). In the DRC, the study was approved by the Kinshasa School of Public Health Ethics Committee. In Nigeria, the study was approved by National Health Research Ethics Committee and in Senegal by the National Committee of Ethics and Research. The research protocols and data collection tools were written in English and translated into French for use in the Francophone countries (DRC and Senegal) to ease data collection and align with country administrative and cultural requirements.

## Results

### Study countries

The DRC and Nigeria are considerably larger in geographical area compared with Senegal and Uganda, and all 4 countries have relatively young populations. The DRC has a population of 85 million people with 52% of the population under the age of 15 years. The proportion of the population below 30 years represents 75% while that of elderly people (60 years and over) is 4% [18]. Nigeria has a population of 206 million people of whom 46% are aged 14 years and below, 50% are aged 15 to 64 years and 4% are aged 65 years and above [19]. The total population in Senegal is 16.7 million people of whom 41.2% are 14 years old and below, 56% aged 15–64 years and 3.0% aged 65 years and older [20]. Uganda has a population of 41.6 million people of which 53% are less than 18 years [21]. Adolescents and youth (10–24 years) are 35% while 4% (1.7 million) of the population are older than 60 years [22].

### Private sector engagement in the COVID-19 response across the four countries

The private sector, both not-for-profit and for-profit entities, participated in COVID-19 strategic preparedness and response operations including strengthening national laboratory systems, treatment and management of COVID-19 cases, risk communication and health promotion and supporting the continuity of access to health services among others, at both national and sub-national levels. In addition, health and non-health private sectors including civil society organizations, individuals, non-governmental organizations and media houses including individuals participated in the COVID-19 response in the four selected countries. The engagement of the private sector can be broadly categorized as support by private for-profit and private not-for-profit health and non-health organizations (Table 1).

**Table 1:** Typology of private sector actors engaging in the COVID-19 pandemic response across the DRC, Nigeria, Senegal and Uganda

	**DRC**	**Nigeria**	**Senegal**	**Uganda**
Private for-profit				
Health	• Laboratory testing companies • Hospitals & clinics, • Manufacturers of therapeutics	• Laboratory testing companies • Hospitals & clinics, • Ambulatory service companies	• Laboratory testing companies • Hospitals & clinics, • Rapid test manufacturing companies • Personal Protective Equipment (PPE) manufacturing companies • Hand sanitizer manufacturers	• Laboratory testing companies • Hospitals & clinics • Rapid test manufacturing companies • Hotels for quarantine & isolation
Non-Health	• Telecom companies		• Financial institutions e.g. banks	• Telecom companies • Media companies • Manufacturing companies • Transport companies • Ambulance service companies
Private not-for-profit				
Health	• Hospitals • Health information system companies such as Bluesquare • Laboratory testing companies • Multilateral organizations, e.g., WB, WHO, UNICEF, CDC	• Coalitions, e.g., CACOVID, • Multilateral organizations e.g. WHO, UNICEF, US CDC	• Multilateral organizations e.g., WHO, UNICEF, US CDC	• Multilateral organizations e.g., WHO, UNICEF, US CDC • Hospitals • Civil Society Organizations
Non- health		• Donations by private individuals, Private foundations e.g., Kensington Adebutu Foundation • Religious institutions		• Associations e.g., Uganda Bankers Association • Private individuals providing food, IPC materials, transport for the sick

### Private sector partnership in surveillance and access to testing services

In the DRC and Nigeria, private entities supported the surveillance activities of the COVID-19 response. In the DRC, in June 2020, a Belgian company (Bluesquare) that provides services for digitizing health systems partnered with the Ministry of Health to implement an e-surveillance system using the existing national surveillance system (District Health Information System). The system incorporated COVID-19 indicators into DHIS-2 and improved timeliness of reporting of these indicators [23]. This process involved the development and validation of the electronic version of the data collection tools used for COVID-19 surveillance, configuration of the adopted COVID-19 data collection tools on mobile phones, computers and testing of the two systems in some health zones. In Nigeria the private laboratories were involved in tracking and re-testing travellers /returnees after quarantine.

Across the 4 countries, the private sector supported expansion of access to testing services. At the beginning of the pandemic in DRC (June 2020), two laboratories located in Kinshasa (HJ Hospital and Centre Médical Diamant) were commissioned to provide free COVID-19 testing services. The Technical Secretariat through the National Biomedical Research Institute (NBRI) beginning June 2020, regularly supplied these laboratories with testing kits and other essential commodities. The cost of a COVID-19 test in DRC ranged between $55 to $75 per test. In Senegal, private laboratories provided testing services at about $42 per test. In Nigeria, private sector laboratories expanded access to testing services through increasing the number of sample collection and testing sites, and they levied a fee of about $125. The use of both private and public laboratories was able to increase the testing throughput in Nigeria to about 15,000—20,000 samples daily by January 2021. In Uganda, at the beginning of the pandemic, all testing services were conducted by the government national laboratories. By October 2020, only symptomatic cases and contacts of confirmed COVID-19 cases were tested by government for free while those that did not fulfil the testing criteria paid $65 equivalent to be tested at private laboratories. This strategy provided a suitable option for individuals who wished to be tested but were ineligible for free testing such as travellers. In Uganda, all COVID-19 private sector testing laboratories went through a certification or accreditation process prior to provision of testing services to the public. The Uganda Ministry of Health (MoH) received applications from private laboratories for accreditation and have the laboratories assessed by a team of experts to ascertain their capacities (i.e., in human resources capacity, equipment availability, quality assurance, standard operating procedures). Furthermore, the MoH required two staff from the private laboratory to be trained at the national reference laboratory. Following formal training these trainees received a panel of 20 samples (5 positives and 15 negatives) to run in their laboratories and the results verified by the national reference laboratory before they were certified to conduct COVID-19 tests. Similarly, in Nigeria, the MoH accredited and approved 36 private laboratories to provide testing services at a fee to international travellers who required a negative polymerase chain reaction (PCR) test result before travelling. Leveraging and partnering with the private laboratories had its own challenges. In the DRC, there were gaps in oversight and quality assurance of the laboratory testing by the private sector. Some private laboratories failed to fully comply with the testing algorithm by using rapid diagnostic tests before their certification which led to the termination of the collaboration by the National Biomedical Research Institute (NBRI). Another challenge related to the engagement of the private sector in the provision of testing services was low reporting of the cases in the national surveillance system. For example, in Uganda, there was  poor and/ or non-reporting of cases from providers of private health services [24, 25] including laboratory testing data into the national information system.

In Senegal and Uganda, Ministries of Health partnered with the private sector to increase access to testing services through establishing partnerships for manufacturing COVID-19 rapid diagnostic tests. In Senegal, a novel rapid diagnostic test (RDT) platform (DiaTropix) was launched by Pasteur Institute in November 2020 to support manufacture of diagnostics for several diseases including COVID-19 antigen RDTs [26, 27]. DiaTropix is a private non-profit company for manufacturing RDTs. In collaboration with the Mérieux Foundation, the Foundation for Innovative and New Diagnostics (FIND), the companies manufactured RDTs to detect COVID-19. By July 31^st^ 2021, 1000,000 test kits had been produced of which 50,000 had already been delivered to the Ministry of Health. In Uganda, the government established a partnership with Astel Diagnostics; a private company to produce RDTs. The RDTs were launched on March 18^th^, 2021 by Makerere University. Development of the kit was supported through public and private sectors including the Government of Uganda, Makerere University through the Research and Innovations Fund, the French Embassy in Uganda, the Uganda Bankers Association, and Astel Diagnostics Uganda, a WHO certified manufacturer.

### Treatment and management of COVID-19

Prior to the COVID-19 pandemic, the government of the Democratic Republic of Congo successfully partnered with Pharmakina, a local manufacturer of malaria therapeutics, to produce the chloroquine and the hydroxychloroquine for COVID-19 case management.

In Nigeria, the Coalition Against COVID-19 (CACOVID) partnership between the private sector and the Federal Government of Nigeria, and the Nigeria Centre for Disease Control (NCDC) and the WHO provided and equipped medical facilities in the six geopolitical zones in Nigeria. The partnership created testing, isolation and treatment centers, provided Intensive Care Units (ICUs) and molecular testing laboratories. Some key informants had this to say:

*“The response gets funds, (this included) funds from the Government, from private sectors, from individuals and also from different partners and NGOs.” (*KII-6, Member of Essential Health Services Coordinator in Nigeria at National Level)

In Nigeria and DRC, other areas of support by the private sector in the COVID-19 response were in the manufacture of disinfectants and personal protective equipment including masks, face shields and hand sanitizers, provision of space for quarantine and isolation and COVID-19 case management and treatment as noted below by a key informant in the DRC.

*“Some brewing companies provided water and alcohol disinfectants to health facilities including to non-COVID essential health services in Kinshasa during the state of emergency period.”* [KII-4, Nurse, Kinshasa University Teaching Hospital]

In Nigeria, local non-governmental organizations such as the Kesington Adebutu Foundation (KAF) donated infection prevention and control commodities including personal protective equipment and ambulances to state health facilities.

In Uganda, the Ministry of Health partnered with owners of privately owned hospitality establishments to provide quarantine services. Private oil and alcohol beverage companies also donated infection prevention and control commodities, ambulances and oxygen to government health facilities.

### Risk communication and health promotion

In the DRC, findings from key informant interviews showed that the private sector engaged in communication to the public through social media to promote access to health services.

*“A private health facility, HJ Hospital, which had observed a 50% decrease in its health services use following state of emergency period and because the population had fear to be infected by COVID-19 at the hospital, decided to use social media (Facebook, Youtube, etc) to promote prompt care-seeking behavior and to raise public awareness about strong Infection Prevention and Control (IPC) measures put in place at the health facility to avoid risk of contamination.”* KII-6, Management Officer, Kinshasa Health Provincial Office

In addition, telecommunication companies were involved in health promotion and risk communication through sending short message services (SMS) on the importance of seeking health services promptly for those that might have symptoms. In addition, messages were also directed to the mothers of children under five years of age for promoting use of routine immunization services. In Uganda, sensitizations about COVID-19 and the importance of infection prevention were supported by a number of private media companies. For example, a Cement Company provided megaphones to be used by community health workers to conduct COVID-19 sensitizations in their communities. Media companies provided free airtime for health workers to conduct radio talk shows to address fears about COVID-19 in the community. Another example was the *“tosemberera “* campaign which translates “do not come near me” was a multi-lingual information campaign coined to promote social distancing to curb transmission of the virus from person to person. Media companies worked jointly to ensure consistent messages like testimonies of people who recovered during the first wave of COVID-19. The risk communication messages emphasized social distancing, avoiding crowds, wearing of masks and washing hands using soap and water or sanitizing hands.

### Supporting continuity of access to other health services

The private sector supported the continuity of access to essential health services by providing essential health services such as maternal and child health services during the pandemic, provision of transport to health workers, establishment of digital platforms to allow access to tele-laboratory and tele-pharmacy services [28] among others. The role of the private sector in ensuring continuity of access to health services was stated by several key informants:

*“The private sector has been pivotal to providing essential health services to people generally. The government facilities can never be enough and you find out in most places there are more private facilities than public facilities… The private facilities are beginning to serve as treatment centers for COVID-19... They have played a very big role in providing essential health services”* (KII-12, State Epidemiology Officer, at State Level)

The private sector utilized telemedicine and other digital health solutions to fill the service delivery gap caused by patients' fears of accessing public facilities [29]. This involved the provision of tele-consultation and tele-psychiatry services.

In Senegal, private companies were engaged in the COVID-19 response through corporate social responsibility activities. For example, Societe Generale Banques au Senegale (SGBS) and Ecobank supported the Mermoz Health Post in a district of Dakar by donating infection prevention and control material such as face masks, soap, and alcohol-based sanitizers. In the medical regions, the association of private doctors (Private Sector Alliance, ASPS) supported the response and played a major role in the response to COVID-19 and maintenance of services through provision of health workers.

In Uganda, the private sector engaged in the COVID-19 response through provision of transport for both patients and health workers. In Tororo, a district in Eastern Uganda, for example, a private bus company donated transport services to transport patients to health facilities as well as transportation for health workers. Furthermore, some private partners donated PPEs, sanitation materials, fuel and food. One of the key informants noted:

*“…We realized that there was a big challenge of transport bringing health workers to work and also bringing patients because of restrictions on use of motor bikes to carry passengers… we worked with a local bus company which provided buses to transport patients and health workers.” KII Six, General Hospital (Uganda)*

In Tororo District in Uganda, individuals including church leaders, health inspectors offered their cars to support movement of mothers to health facilities for deliveries and other emergency services in the communities.

In Lamwo District in Uganda, health workers in private clinics were trained to offer quality services such malaria, pneumonia and identification of danger signs for pregnancy and to make appropriate referral especially for pregnant mothers. Regarding supplies and commodities, private pharmacies enabled people to buy personal protective equipment (PPEs) and filled in widespread stockouts. Private pharmacists also provided medicines in the remote areas in the Northern and West Nile regions of the country.

Private companies also provided physical cash to the districts and National task force to support the COVID-19 response. Donations like food also helped staff remain at the station to ensure the continuity of essential services.

## Discussion

The private sector engaged in the COVID-19 response in DRC, Nigeria, Senegal and Uganda included both the for-profit and the not-for-profit entities. The private-for-profit entities were those with products and services that directly support the response such as providers of testing and case management services. The not-for-profit private sector included entities that participated as part of corporate social responsibility and international multi-lateral organizations such as WHO, UNICEF and other civil society organizations. Efforts to fully leverage the private sector should be cognizant of the various sub-categories within the private sector and the variation in objectives and motives of each entity so that partnerships are mutually beneficial and sustainable.

The engagement of the private sector in the COVID-19 response in the DRC, Nigeria, Senegal, and Uganda can be categorized into four broad themes, namely COVID-19 surveillance and access to testing, management of cases for COVID-19, conducting risk communication and health promotion and continuity of access to other non-COVID-19 essential services. Across the four countries, governments leveraged the support from the private sector to expand access to testing services for people who needed testing services but did not fulfil the testing criteria such as international travellers. Although testing services were provided at a fee by the private sector, it reduced the demand for testing services that had to be provided by the public sector for the large numbers of international travellers. Expansion of testing services to the private sector was met by several challenges. First, of the four countries under study, only Uganda and Nigeria reported explicit procedures of quality assessment prior to being accredited to conduct COVID-19 tests. Accreditation verifies the implementation of accepted laboratory standards [30], minimizes the variability of test results and reduces the frequency of errors [31]. Countries need to establish procedures for certifying private laboratories to conduct COVID-19 testing. The procedures should start with accreditation, followed by regular auditing and inspection to maintain quality and sustain the partnerships. These procedures failed in DRC because private laboratories did not comply with the recommended testing algorithm which led to the termination of the partnerships with some private sector providers. The second challenge is regulation of service fees for COVID-19 tests, which were not affordable for most of the population. For example, in Uganda, the price for COVID-19 tests by private providers were as high as $65 per test, $125 per test in Nigeria, between $55 to $75 per test, depending on the private sector provider in DRC and $42 in Senegal. Testing for COVID-19 is so critical in the COVID-19 response that some researchers have recommended regular free mass testing to contain the spread of the virus [32] and is one of the explanatory factors for the exemplary performance of South Korea [33] and Vietnam [34] in containing the spread of COVID-19. Countries should develop partnerships and frameworks for the engagement of the private sector at national and international level to reduce the price of testing and increase the demand for testing services. This was exemplified by state institutions in Senegal and Uganda who partnered with international companies to provide COVID-19 rapid diagnostic tests.

Across the four countries, the private sector was also engaged in the treatment and management of COVID-19 cases. However, there was an additional challenge of poor reporting of the services provided by the private sector into the national surveillance system particularly in Uganda. There is a need for a conducive policy environment that a) promotes and encourages involvement in emergency preparedness and response b) promotes alignment with national guidelines and requirements like reporting while c) assuring the quality of the services provided. Governments can establish partnerships with the private sector that will facilitate subsidising the cost of managing COVID-19 patients in private health facilities.

Across the four countries, the private sector including individuals and manufacturing companies provided infection prevention and control commodities such as personal protective equipment, gloves, aprons, and hand sanitizers. Infection prevention and control is indeed critical to robust preparedness and response strategies and was highlighted as one of the response operations by the WHO COVID-19 strategic preparedness and response plan [16] for member states in order to ensure the safety of health workers and population at large.

However, the COVID-19 strategic preparedness and response plan also included operations such as risk communication and community engagement, surveillance, points of entry (passages for international entry and exit of travellers and cargo), rapid response teams, national laboratory systems, case management, continuity of essential health services and logistics, procurement and supply management. Albeit having this framework on response, there was a lack of guidance on which areas and how to engage and optimize the private sector in the response operations to mount effective and efficiently coordinated responses. According to the Global Health Security Index [9] where private sector engagement in disease preparedness and response is under the capacity on emergency preparedness and response, the Democratic Republic of Congo scored 25%, Nigeria scored 12.5%, Senegal scored 12.5% while Uganda scored 0%. The WHO Joint External Evaluation of DRC [35] noted that although the private sector engages in disease surveillance and response, it is vague about the specific ways in which it participates. In Nigeria, the Nigeria Centers for Disease Control (NCDC) in partnership with the Private Sector Health Alliance of Nigeria (PHN) launched the Alliance for Epidemic Preparedness and Control in 2018 to facilitate the formal engagement of the country’s private sector in the preparedness, response and control of public health emergencies [36]. However, by 2019, the Global Health Security Index noted that the initiative was yet to be operationalized [9]. In addition, the index noted that although both Senegal and Uganda had documents that had reference to private sector engagement, e.g., the National Malaria Strategic Plan [37] in Senegal and the National Policy for Disaster Preparedness and Management [38] in Uganda, there was no evidence of a policy framework to guide the engagement of the private sector in disease preparedness, response and control [9].

Noteworthy is that although there was significant material and infrastructural support for the COVID-19 response from the private sector, there was little direct support of the human resources by this sector across the 4 countries. It is only in Senegal where the association of private health care workers provided human resources to support the treatment and management of COVID-19 cases. Furthermore, capacity building initiatives such as mentoring, coaching and support supervision in the health sector should include health workers in public and private facilities to strengthen and sustain partnerships between the public and private sectors. In Uganda, media companies provided free airtime for District Health Officers to communicate about COVID-19 and continuity of essential health services such as maternal and child health services. Any guiding framework on engaging the private sector should outline ways in which private mobilization and community engagement expertise in the communication of the COVID-19 prevention messages can be fully leveraged. The framework that formalizes the engagement can clarify how the support need not be material only but should aim to leverage established infrastructure of the private entities as was done by telecommunication companies in Iran [39]. A starting point for the development of these guiding documents is the WHO Action Plan for Engagement of the Private Health Sector in the COVID-19 response which categorized the engagement into space (infrastructure and facilities), staff (numbers and cadres), stuff (equipment, testing, PPE, supplies), systems (data, communication, transport) and supply side financing [40]. In addition to the private health sector engagement, the private–public sector partnership framework should outline how other segments of the private sectors beyond health can contribute to national priorities for the response and control of public health emergencies in general, and the COVID-19 pandemic in particular.

### Limitations

The results and interpretations of our findings are subject to several limitations. First, we conducted the work in the middle of a pandemic which might have limited access to some important government policies and documents. Secondly, the response to the COVID-19 pandemic has been evolving and these findings are not comprehensive as they only capture the response during the peak of the pandemic. Similarly, the transferability of learnings and lessons is limited to countries that have similar health system contexts where the private sector plays a significant role in the provision of health services. However, the findings have significant utility currently as countries continue to respond to new waves of the COVID-19 pandemic.

## Conclusions

The private sector has contributed to the COVID-19 response through engagement in COVID-19 surveillance and testing, treatment and management of COVID-19 cases, risk communication and health promotion, and maintenance of access to other essential services. However, there is a need to further harness the public–private partnerships by developing comprehensive and evidenced-based regulatory frameworks and policy environments that promote engagement in operations of the response such as communication and community engagement, vaccination and procurement and supply management. Furthermore, the regulatory frameworks should ensure that the contribution of the private sector is aligned to national strategies and priorities and should include issues such as pricing, assurance and maintenance of the quality of services provided by the private sector.

## Data Availability

The dataset used for analysis can be availed upon reasonable request by writing an email to the corresponding author.

## Abbreviations

COVID-19: Corona Virus Disease 2019
DRC: Democratic Republic of Congo
GHSI: Global Health Security Index
INRB: National Biomedical Research Institute (DRC)
LMIC: Low and Middle Income Country
RDT: Rapid Diagnostic Test
WHO: World Health Organization
